# Effects of renin-angiotensin system inhibitor and beta-blocker use on mortality in older patients with heart failure with reduced ejection fraction in Japan

**DOI:** 10.3389/fcvm.2024.1377228

**Published:** 2024-05-31

**Authors:** Kei Kawada, Tomoaki Ishida, Hitoshi Fukuda, Yuki Hyohdoh, Toru Kubo, Tomoyuki Hamada, Yuichi Baba, Toshinobu Hayashi, Fuka Aizawa, Kenta Yagi, Yuki Izawa-Ishizawa, Takahiro Niimura, Shinji Abe, Mitsuhiro Goda, Hiroaki Kitaoka, Keisuke Ishizawa

**Affiliations:** ^1^ Department of Clinical Pharmacy Practice Pedagogy, Tokushima University Graduate School of Biomedical Sciences, Tokushima, Japan; ^2^ Department of Clinical Pharmacology and Therapeutics, Tokushima University Graduate School of Biomedical Sciences, Tokushima, Japan; ^3^ Department of Pharmacy, Kochi Medical School Hospital, Kochi, Japan; ^4^ Department of Neurosurgery, Kochi Medical School, Kochi University, Nankoku, Japan; ^5^ Center of Medical Information Science, Kochi Medical School, Kochi University, Kochi, Japan; ^6^ Department of Cardiology and Geriatrics, Kochi Medical School, Kochi University, Kochi, Japan; ^7^ Department of Emergency and Disaster Medical, Pharmacy Faculty of Pharmaceutical Sciences, Fukuoka University, Fukuoka, Japan; ^8^ Department of Pharmacy, Tokushima University Hospital, Tokushima, Japan; ^9^ Clinical Research Center for Developmental Therapeutics, Tokushima University Hospital, Tokushima, Japan; ^10^ Department of General Medicine, Taoka Hospital, Tokushima, Japan

**Keywords:** heart failure, medications, beta-blockers, angiotensin-converting enzyme inhibitors, angiotensin II receptor blockers, heart failure with reduced left ventricular ejection fraction, older patients with heart failure

## Abstract

**Introduction:**

Guideline-directed medical therapy with renin-angiotensin system (RAS) inhibitors and beta-blockers has improved the survival of patients with heart failure (HF) and reduced left ventricular ejection fraction (HFrEF). However, it is unclear whether RAS inhibitors and beta-blockers can be administered to older patients with HF. Therefore, this study aimed to investigate the effects of beta-blockers and RAS inhibitors on the prognosis of older patients with HFrEF.

**Methods:**

Demographic, clinical, and pharmacological data from 1,061 patients with acute decompensated HF, enrolled in the Kochi Registry of Subjects with Acute Decompensated Heart Failure (Kochi YOSACOI study), were analyzed to assess their impact on mortality. Additionally, a machine learning approach was applied to complement the conventional statistical model for analysis. Patients with HFrEF (*n* = 314) were divided into the all-cause mortality within 2 years group (*n* = 80) and the survivor group (*n* = 234).

**Results:**

Overall, 41.1% (129/314) of the patients were aged ≥80, and 25.5% (80/314) experienced all-cause mortality within 2 years. Furthermore, 57.6% (181/314) and 79.0% (248/314) were prescribed RAS inhibitors and beta-blockers, respectively. Our analysis showed that RAS inhibitor use was associated with reduced all-cause mortality and cardiac death in patients with HFrEF of all ages (*P* < 0.001), and beta-blocker use had an interaction with age. Machine learning revealed that the use of beta-blockers altered the risk of mortality, with a threshold of approximately 80 years of age. Beta-blocker use was associated with lower all-cause mortality and cardiac death in patients with HFrEF aged <80 years (*P* < 0.001) but not in those aged ≥80 years (*P* = 0.319 and *P* = 0.246, respectively). These results suggest that beta blockers may differ in their all-cause mortality benefits according to age.

**Conclusions:**

RAS inhibitors prevented all-cause mortality and cardiac death at all ages, whereas beta-blockers had different effects depending on the patient's age. This study suggested that the choice of beta-blockers and RAS inhibitors is more important in older patients with HFrEF than in younger patients with the same condition.

## 1 Introduction

Heart failure (HF) places a considerable economic burden on healthcare systems worldwide owing to its high morbidity and mortality, as well as frequent and prolonged hospitalizations (1–5). Because the prevalence of HF increases with age, the incidence of patients with HF is continually increasing with an aging society (6, 7). In addition, increased life expectancy and improved management of acute cardiac events and complications have led to a steady increase in the age of patients with HF and reduced left ventricular ejection fraction (HFrEF) (8, 9).

Angiotensin-converting enzyme inhibitors, angiotensin II receptor blockers, beta-blockers, and mineralocorticoid receptor antagonists (MRAs) are the mainstays of guideline-directed medical therapy for patients with HFrEF (10–12). Clinical trials have shown that angiotensin receptor neprilysin inhibitors (ARNI) and sodium-glucose cotransporter (SGLT) 2 inhibitors reduce the risk of HF hospitalization and death (13–15). Recent guidelines recommend using ARNI and SGLT2 inhibitors (12, 16). Large clinical trials have demonstrated that beta-blockers and renin-angiotensin system (RAS) inhibitors improve the prognosis of patients with HFrEF (12, 16). However, these findings were obtained from HF patients younger than real-world patients, as they are the results of randomized clinical trials that excluded older HF patients with comorbidities, functional and cognitive impairments, and a poor prognosis due to increased polypharmacy. Therefore, whether these results are directly applicable to older patients with HF is unclear (17).

This study aimed to investigate the effects of beta-blockers and RAS inhibitors on the prognosis of older patients with HFrEF. Using data from the Kochi Registry of Subjects with Acute Decompensated Heart Failure (Kochi YOSACOI study), we applied novel game theory-based methods in explainable machine learning (ML) to identify factors associated with all-cause mortality within 2 years in older patients with HFrEF in addition to traditional statistical analyses models.

## 2 Materials and methods

### 2.1 Patient population

We used data from the Kochi YOSACOI study, which enrolled 1,061 consecutive patients with acute decompensated HF (ADHF) in Kochi, Japan, from May 2017–December 2019. In addition, we used data on clinical outcomes for all-cause mortality within 2 years with follow-ups through December 2021. Details of the Kochi YOSACOI study have been described previously (18). Briefly, the Kochi YOSACOI study was a collaborative effort of six hospitals that provide acute care for cardiovascular diseases in Kochi Prefecture, where the percentage of people over 65 years of age has reached 35%. All participating hospitals practiced acute HF treatment according to standard guidelines (11). Eligibility criteria for inclusion in the registry were age ≥20 years and hospitalization for ADHF at one of the participating hospitals. Based on the Framingham criteria, ADHF was diagnosed by the presence of at least two major criteria, including symptoms and physical examination, chest radiography, and echocardiographic findings, or the presence of one major and two minor criteria.

This study was approved by the Medical Research Ethics Committee of Kochi University of Medical Science (Approval No. 28–68) and the Medical Research Ethics Committee of Tokushima University Graduate School of Biomedical Sciences (Approval No. Z120). The study complied with the tenets of the Declaration of Helsinki, and informed consent was obtained from all patients or their families. Confidentiality and anonymity of patient data were maintained throughout.

### 2.2 Patient selection and study design

The clinical characteristics of the patients have been described previously in detail (18). Data were collected by investigators at participating hospitals during the enrollment period. We obtained information on patient demographics, etiology of HF, medical history, long-term treatment, HF symptoms and vital signs at admission and discharge, discharge prescriptions, laboratory and echocardiographic data, and other clinical parameters. We used echocardiographic data from the time when HF status was stabilized during hospitalization. After HF stabilization, left ventricular ejection fraction (LVEF) was echocardiographically determined during hospitalization, and HF with an LVEF of ≤40% was categorized as HFrEF. Patients' nutritional status was assessed using the Geriatric Nutritional Risk Index (GNRI), a simple measure of nutritional status in older adults, calculated using the following equation: GNRI = 14.89 × serum albumin (g/dl) + 41.7 × body mass index/22 (19). RAS inhibitor use was defined by the prescription of angiotensin-converting enzyme inhibitors or angiotensin II receptor blockers. The RAS inhibitor and beta-blocker use was defined as patients receiving a prescription for RAS inhibitor (enalapril, lisinopril, and candesartan) and beta-blockers (carvedilol and bisoprolol) at the time of discharge. Because patients with HFrEF in this study were mainly older adults, MRAs were assumed to be used less frequently because of the risk of adverse events due to hyperkalemia when MRAs are added to RAS inhibitors. Therefore, we did not include MRAs in this study. In addition, ARNI and SGLT 2 inhibitors were not approved in Japan during the enrollment period (20).

**Figure 1:**
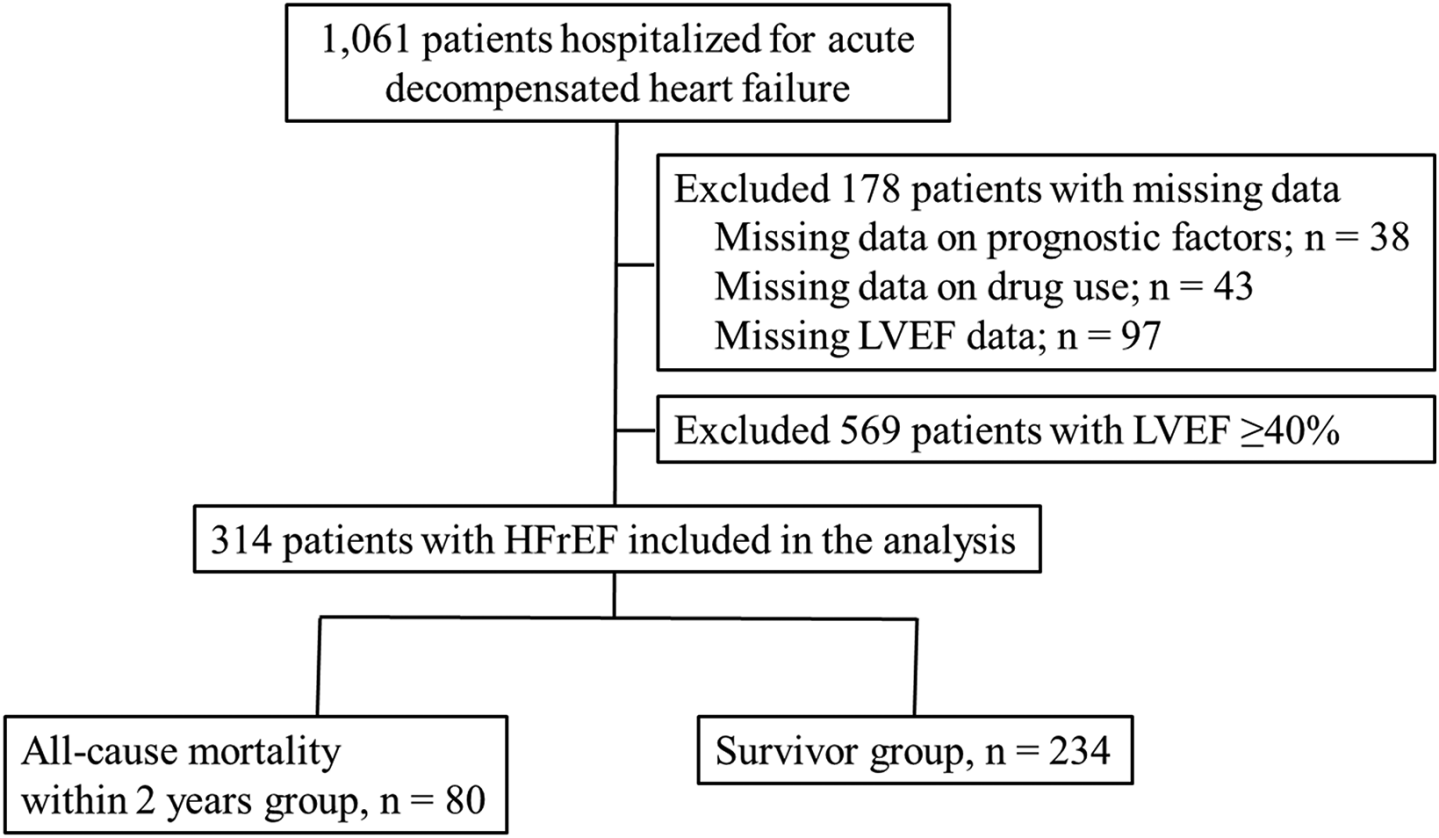
Study flow chart. HFrEF, heart failure with reduced left ventricular ejection fraction; LVEF, left ventricular ejection fraction.

Of the patient cohort (*n* = 1,061), we excluded 38 patients with missing all-cause mortality data within 2 years, 43 with missing drug data, and 97 with missing LVEF data at admission. Of the remaining 883 patients, all patients with HFrEF (*n* = 314) were included and divided into two groups according to all-cause mortality within 2 years: the all-cause mortality within 2 years group (*n* = 80) and the survivor group (*n* = 234; Figure 1).

We performed univariate and multivariate analyses of the association between RAS inhibitor and beta-blocker use and 2-year mortality. The interaction of RAS inhibitor and beta-blocker use with age was also analyzed to determine whether the efficacy of these medications depends on age. Additionally, all patients with HFrEF (*n* = 314) were divided into two groups according to cardiac death within 2 years: cardiac death within 2-year group (*n* = 41) and survival group (*n* = 273). Furthermore, Kaplan–Meier survival analysis was used to assess the effects of RAS inhibitors and beta-blockers on 2-year all-cause mortality and 2-year cardiac mortality, respectively, stratified by age.

### 2.3 Statistical analysis

All data are expressed as medians with interquartile ranges (IQRs) for non-normally distributed variables or frequencies (percentages) for categorical data. Differences in continuous variables were assessed using Student's *t*-test or the Mann–Whitney *U* test. Pearson's chi-squared test was used to compare categorical variables, and Fisher's exact test was used when the expected frequency was <5. Hazard ratios (HRs) and 95% confidence intervals (CIs) were also determined using logistic regression analysis. A *P*-value < 0.05 was indicated significance for all tests. Cumulative event rates were assessed using the Kaplan–Meier method. Multivariate Cox regression analysis was used to evaluate the adjusted relative risk of the variables. To investigate the nonlinear associations and interactions among risk factors associated with all-cause mortality in patients with HFrEF via an ML model, we used Light Gradient Boosting Machine (LightGBM), a variant of Gradient Boosting Decision Trees (GBDTs) (21). ML techniques offer the advantage of enabling the identification of important variables, including potentially obscured variable interactions and data patterns that contribute to patient outcomes. These may not be easily discovered by researchers using traditional statistical approaches (22). We developed a mortality prediction model for patients with HFrEF using previously described variables as explanatory variables and assessed the occurrence of all-cause mortality as the object variable. ML models are known for their high predictive accuracy, but they can also overfit. Overfitting occurs when a model performs well on the data used to develop it but fails to predict unseen data. To mitigate this risk, evaluating the model's performance on an independent dataset is important. Therefore, when building the mortality prediction model, we randomly allocated 80% of the dataset for model development and reserved the remaining 20% for validation. We evaluated the ML models using accuracy verification data and confirmed that the model in this study was unlikely to be overfitting. We used Shapley additive explanations (SHAP) values to quantify the influence of interactions between features (23). The primary focus of this analysis was the interaction between age and other variables. Using SHAP interaction plots, we explicitly showed how age and the variables of interest affect predictions and explored interaction effects that might modify the mortality risk associated with age. Statistical analyses were conducted using the R (version 3.3.1, R Foundation for Statistical Computing, Vienna, Austria. http://www.Rproject.org) and Python (version 3.8.10, Python Software Foundation, https://www.python.org) software.

## 3 Results

### 3.1 Patient characteristics

We analyzed 314 patients with HFrEF. The median age was 76 (IQR, 68.0–83.0) years, 41.1% (129/314) were aged ≥80, 57.6% (181/314) were prescribed RAS inhibitors, and 79.0% (248/314) were prescribed beta-blockers (Table 1). Moreover, 25.5% (80/314) experienced all-cause mortality within 2 years. Patients in the all-cause mortality group had a higher age, lower GNRI, higher rate of New York Heart Association (*N*YHA) class III/IV at discharge, higher rate of a prior HF-related admission, and higher rate of chronic obstructive pulmonary disease (COPD) than did patients in the survivor group. Patients in the all-cause mortality group had lower estimated glomerular filtration rate (eGFR) and sodium levels but higher B-type natriuretic peptide (BNP) levels than did patients in the survivor group. Patients in the all-cause mortality group had lower proportions of RAS inhibitor (35% vs. 65.4%; *P* < 0.001) and beta-blocker (60.0% vs. 85.1%; *P* < 0.001) use than did patients in the survivor group.

**Table 1:** Baseline characteristics of the enrolled patients.

Variable	All patients	No all-cause mortality	All-cause mortality	*P*-value
	(*n* = 314)	(*n* = 234)	(*n* = 80)	
Age (years)	76.0 [68.0, 83.0]	73.5 [65.0, 81.0]	83.0 [74.8, 88.0]	<0.001
Age ≥80	129 (41.1)	83 (35.5)	46 (57.5)	<0.001
Sex, female	118 (37.6)	87 (37.2)	31 (38.9)	0.89
BMI (kg/m^2^)	20.6 [18.3, 23.2]	19.3 [17.2, 21.9]	21.1 [18.9, 23.6]	0.002
GNRI	92.2 [83.6, 100.0]	94.1 [85.5, 101.6]	86.7 [77.3, 94.1]	<0.001
NYHA class III/IV at discharge	16 (5.1)	8 (3.4)	8 (10.0)	0.044
Prior HF-related admission	111 (35.4)	69 (31.6)	36 (47.5)	<0.001
Etiology of heart failure exacerbation				
Ischemic heart disease	118 (37.6)	86 (36.8)	32 (40.0)	0.688
Valvular heart disease	30 (9.6)	17 (7.3)	13 (16.3)	0.026
Cardiomyopathy	101 (32.2)	76 (32.5)	25 (31.3)	0.89
Hypertension	19 (6.1)	18 (7.7)	1 (1.3)	0.053
Comorbidities				
Hypertension	197 (62.7)	150 (64.1)	47 (58.8)	0.42
Diabetes mellitus	110 (35.0)	79 (33.8)	31 (38.8)	0.42
Dyslipidemia	148 (47.1)	107 (45.7)	41 (51.3)	0.44
Atrial fibrillation/flutter	130 (41.4)	94 (40.2)	36 (45.0)	0.51
Old myocardial infarction	72 (22.9)	51 (21.8)	21 (26.2)	0.44
COPD	28 (8.9)	15 (6.4)	13 (16.2)	0.015
Bronchial asthma	16 (5.1)	11 (4.7)	5 (6.3)	0.56
Cerebrovascular accident	57 (18.2)	42 (17.9)	15 (18.8)	0.87
Treatment in the acute phase				
NIPPV/ventilator	47 (15.0)	36 (15.4)	11 (13.8)	0.86
Inotropic agents	63 (20.1)	42 (17.9)	21 (26.3)	0.145
Laboratory data at discharge				
BNP, pg/ml	378.8 [196.2, 621.1]	318.9 [172.0, 554.2]	503.0 [334.5, 888.2]	<0.001
eGFR, ml/min/1.73 m^2^	46.3 [32.6, 62.7]	48.9 [35.4, 64.4]	40.2 [27.0, 56.9]	0.004
Sodium, mEq/L	139 [137, 141]	139.0 [137.0, 141.0]	137 [134.5, 139.5]	<0.001
LVEF, %	30 [25, 35]	30 [24, 36]	30 [25, 35]	0.81
Medication at discharge				
RAS inhibitors	181 (57.6)	153 (65.4)	28 (35)	<0.001
Beta-blockers	248 (79.0)	200 (85.1)	48 (60.0)	<0.001
MRAs	142 (45.2)	106 (45.3)	36 (45.0)	0.99
Loop diuretics	290 (92.4)	223 (95.3)	67 (83.8)	0.002
Thiazide diuretics	18 (5.7)	12 (5.1)	6 (7.5)	0.414
Calcium channel blockers	57 (18.2)	44 (18.8)	13 (16.3)	0.737
Digitalis	1 (0.3)	1 (0.3)	0 (0.0)	0.99
Tolvaptan	90 (28.7)	63 (26.9)	27 (33.8)	0.254
Anticoagulant agents	156 (49.7)	113 (48.3)	43 (53.8)	0.438

BMI, body mass index; BNP, B-type natriuretic peptide; COPD, chronic obstructive pulmonary disease; eGFR, estimated glomerular filtration rate; GNRI, Geriatric Nutritional Risk Index; HF, heart failure; LVEF, left ventricular ejection fraction; MRA, mineralocorticoid receptor antagonist; NIPPV, noninvasive positive pressure ventilation; NYHA, New York Heart Association; RAS, renin-angiotensin system.

Data are shown as median [interquartile range] or *n* (%).

### 3.2 Risk factors for all-cause mortality

We investigated factors related to all-cause mortality in patients with HFrEF (Table 2). The univariate analysis showed that advanced age, GNRI, NYHA class III/IV at discharge, prior HF-related admission, COPD, high BNP levels, low eGFRs, low sodium levels, RAS inhibitor use, and beta-blocker use were associated with all-cause mortality. The multivariate analysis showed that advanced age (adjusted HR, 1.049; 95% CI, 1.022–1.078; *P* < 0.001), prior HF-related admission (adjusted HR, 1.873; 95% CI, 1.160–3.025; *P* = 0.01), and RAS inhibitor use (adjusted HR, 0.436; 95% CI, 0.26–0.729; *P* = 0.002), but not beta-blocker use (adjusted HR, 0.807; 95% CI, 0.464–1.406; *P* = 0.45; Table 2), were independently associated with all-cause mortality.

**Table 2:** Predictors of all-cause death within 2 years in patients with HFrEF.

	Univariate			Multivariate		
	HR	95% CI	*P*-value	Adjusted HR	95% CI	*P*-value
Age (years)	1.067	1.042–1.092	<0.001	1.049	1.022–1.078	<0.001
Sex, female	1.14	0.724–1.794	0.572			
GNRI	0.974	0.961–0.988	<0.001	1	0.979–1.021	0.978
NYHA class III/IV at discharge	3.325	1.594–6.935	0.001	1.927	0.884–4.204	0.099
Prior HF admission	2.518	1.601–3.960	<0.001	1.873	1.160–3.025	0.01
Comorbidities						
Hypertension	0.791	0.505–1.240	0.308			
Diabetes mellitus	1.231	0.783–1.935	0.368			
Dyslipidemia	1.109	0.711–1.731	0.648			
Atrial fibrillation/flutter	1.277	0.818–1.993	0.283			
Old myocardial infarction	1.313	0.794–2.171	0.288			
COPD	1.899	1.013–3.561	0.045	1.827	0.970–3.443	0.062
Bronchial asthma	1.425	0.575–3.529	0.444			
Cerebrovascular accident	1.237	0.703–2.177	0.461			
Laboratory data at discharge						
eGFR, ml/min/1.73 m^2^	0.989	0.978–1.000	0.04	0.999	0.988–1.010	0.833
LVEF, %	0.992	0.960–1.024	0.607			
Medication at discharge						
RAS inhibitors	0.32	0.201–0.507	<0.001	0.436	0.26–0.729	0.002
Beta-blockers	0.374	0.234–0.596	<0.001	0.807	0.464–1.406	0.45

BNP, B-type natriuretic peptide; CI, confidence interval; COPD, chronic obstructive pulmonary disease; eGFR, estimated glomerular filtration rate; GNRI, Geriatric Nutritional Risk Index; HF, heart failure; HFrEF, heart failure with reduced ejection fraction; HR, hazard ratio; LVEF, left ventricular ejection fraction; NYHA, New York Heart Association; RAS, renin-angiotensin system.

### 3.3 Subgroup analyses of age-variable interactions

The variables RAS inhibitor and beta-blocker use were tested for their interactions with the variable age. The interaction between RAS inhibitor use and age was not significant (*P* = 0.814) (Table 3), but that between beta-blocker use and age was significant (*P* = 0.031). The results of the subgroup analyses for the interactions of age showed a differential effect of age on RAS inhibitor and beta-blocker use among patients with HF.

**Table 3:** Subgroup analyses of age–variable interactions.

Age variables	HR	95% CI	*P*-value
RAS inhibitors	1.007	0.951–1.067	0.814
Beta-blockers	1.063	1.006–1.124	0.031

CI, confidence interval; HR, hazard ratio; RAS, renin-angiotensin system.

### 3.4 Predicted all-cause mortality within 2 years

**Figure 2:**
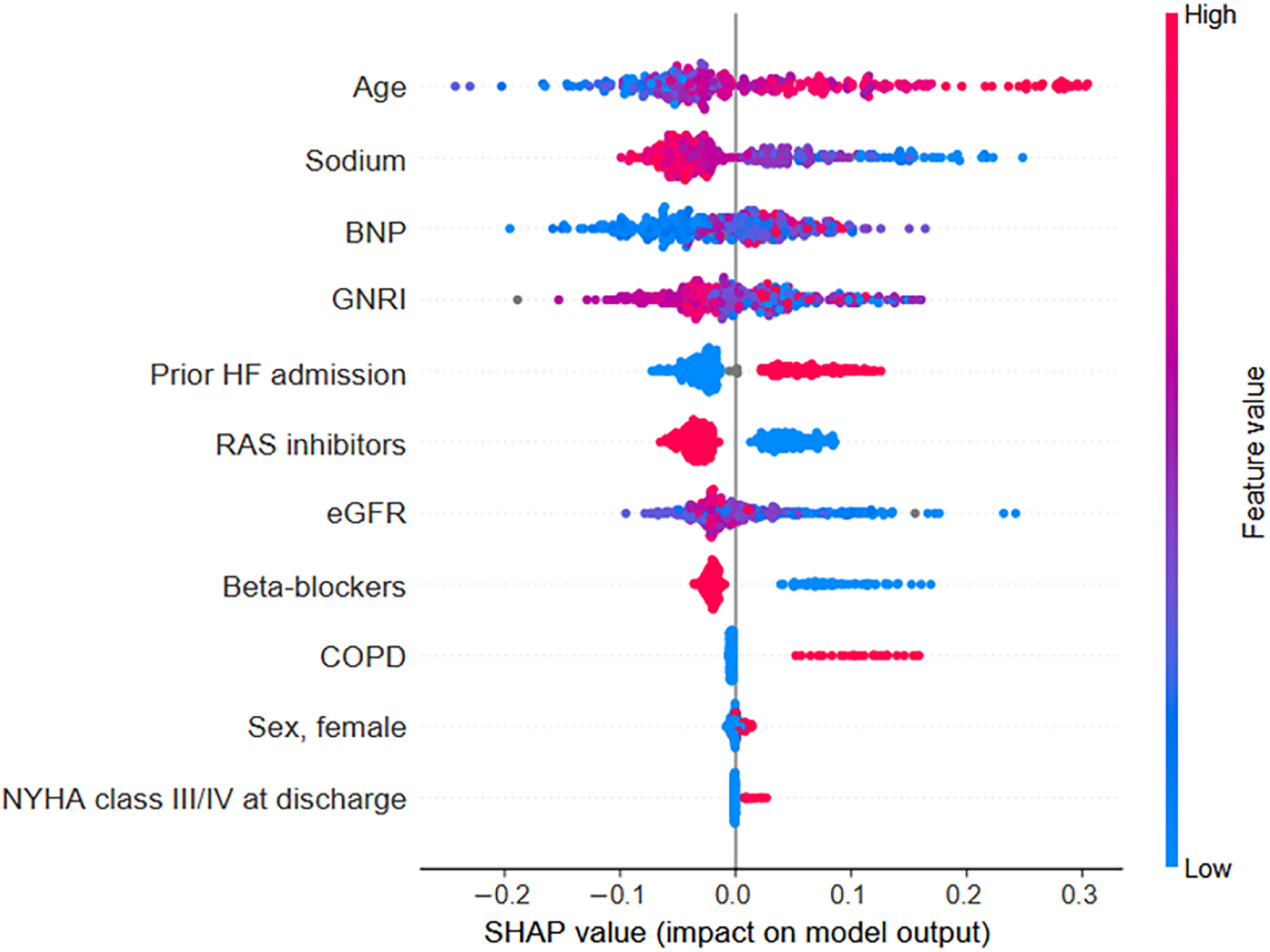
SHAP values for each variable were calculated from the individual values of each patient within the prediction model. Each data point is represented by a dot with a color based on the value of the variable. Red dots indicate high values for a particular patient's variable, whereas blue dots indicate low values. For binary categorical variables, a blue dot indicates the absence of the category, and a red dot indicates its presence. By visualizing these patient-specific SHAP values, the relationship between each variable's value (indicated by the dot color) and its impact on the model's output becomes clear. BNP, B-type natriuretic peptide; COPD, chronic obstructive pulmonary disease; eGFR, estimated glomerular filtration rate; GNRI, Geriatric Nutritional Risk Index; NYHA, New York Heart Association; RAS, renin-angiotensin system; SHAP, Shapley additive explanations.

GBDT models were constructed for each patient to predict the probability of all-cause mortality within 2 years. The GBDT model's performance, evaluated through the Area Under the Curve (AUC) for the validation data, was 0.72 (95% CI, 0.568–0.871). The performance shows that our GBDT models were not vulnerable to overfitting. SHAP values were calculated for each patient-derived model to quantify feature importance and interaction effects. Calculation and ranking of SHAP values for each variable of patient data revealed that older age, low sodium levels, high BNP levels, low GNRI, prior HF-related admission, RAS inhibitor use, and beta-blocker use at discharge were the most important features for a predicted increased all-cause mortality within 2 years across patients with HFrEF (Figure 2).

### 3.5 Interactions between age and beta-blocker use

**Figure 3:**
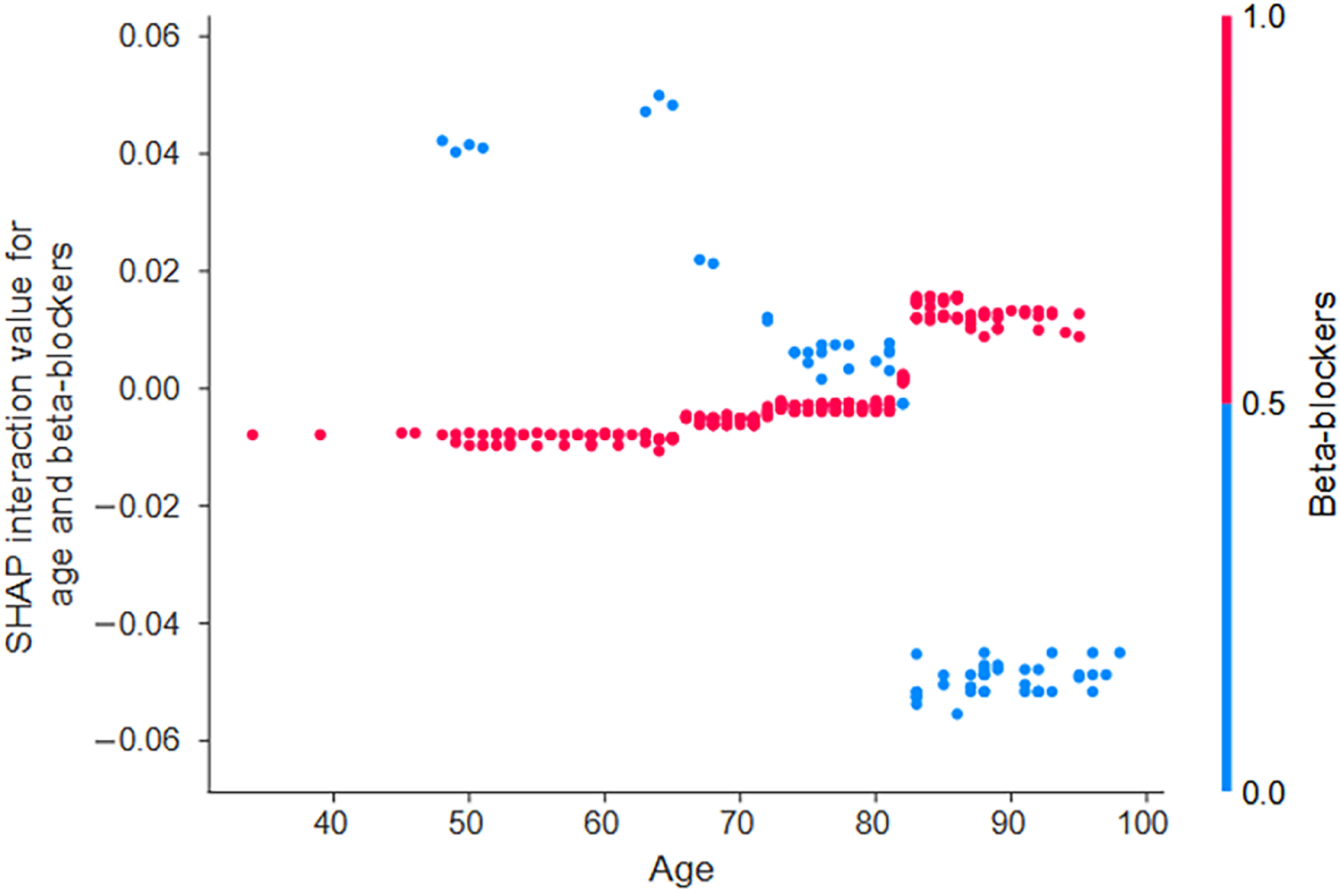
The interaction effects of beta-blockers influence the predicted probability of all-cause mortality based on age. The SHAP interaction values quantify these effects and show the interaction between age and beta-blocker use. When the SHAP interaction values (y-axis) are plotted as a function of patient age (x-axis) and the value of the interacting variable (indicated by the color of the dot—red for beta-blocker use and blue for non-use), trends in variables and their values that have a greater interaction effect emerge. In the case of age, an interaction effect with beta-blocker use becomes apparent at approximately 80 years of age. SHAP, Shapley additive explanations.

When examining the SHAP interaction values, an interaction was noted between age and beta-blocker use (Figure 3). In patients aged 80 years and older, the risk of mortality within 2 years differed depending on whether beta-blockers were used or not. Specifically, in patients over 80 years of age, not using beta-blockers slightly reduced the age-related risk of mortality. Thus, compared with no use, beta-blocker use increased the risk of age-related mortality. In other words, the results of this analysis showed that the use of β-blockers altered the risk of mortality, either by increasing or decreasing it, with a threshold of approximately 80 years of age.

### 3.6 Subgroup analysis of RAS inhibitor and beta-blocker use by patient age

We performed an age-dependent subgroup analysis of the effects of RAS inhibitor and beta-blocker use on all-cause mortality. RAS inhibitor use was associated with a reduced risk in both age groups. By contrast, beta-blocker use was associated with a reduced risk in the age <80 years group (adjusted HR, 0.486; 95% CI, 0.247–0.956; *P* = 0.037), but the risk tended to increase in the age ≥80 years group (adjusted HR, 1.966; 95% CI, 0.97–3.985; *P* = 0.061; Table 4).

**Table 4:** Subgroup analyses by age.

Variables	Adjusted HR	95% CI	*P*-value
RAS inhibitor use			
Age <80 years	0.423	0.181–0.991	0.048
Age ≥80 years	0.223	0.083–0.598	0.003
Beta-blocker use			
Age <80 years	0.486	0.247–0.956	0.037
Age ≥80 years	1.966	0.97–3.985	0.061

CI, confidence interval; HR, hazard ratio; RAS, renin-angiotensin system.

### 3.7 Clinical outcomes

**Figure 4:**
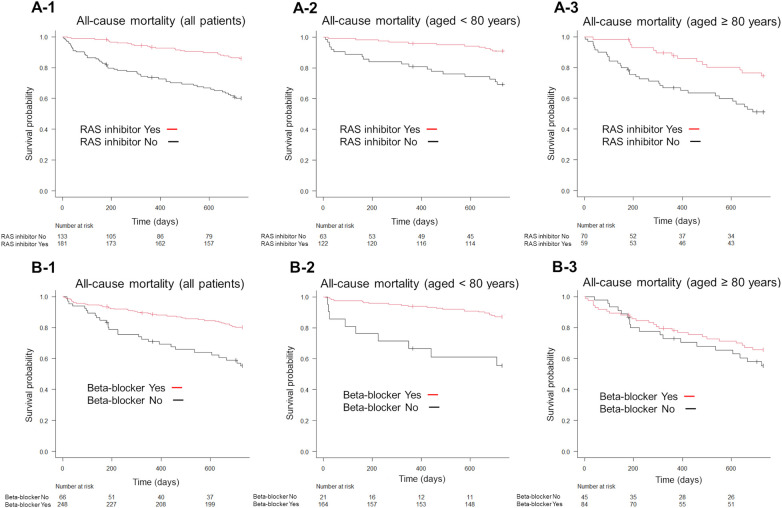
Kaplan–Meier survival curves for the endpoint of all-cause mortality, according to the GDMT strategy. Patients receiving RAS inhibitors exhibited lower all-cause mortality than those not receiving RAS inhibitors (**A-1**). Similarly, patients on beta-blockers exhibited lower all-cause mortality than those not on beta-blockers (**B-1**). The use of RAS inhibitors significantly reduced all-cause mortality in both patient groups, those aged <80 years and those aged ≥80 years, compared to non-use (**A-2,A-3**). In patients aged <80 years, beta-blocker use was associated with lower all-cause mortality than non-use (**B-2**), whereas in patients aged ≥80 years, beta-blocker use did not show a significant effect compared to non-use (**B-3**). GDMT, guideline directed medical therapy; RAS, renin-angiotensin system.

Kaplan–Meier survival analysis showed that RAS inhibitor and beta-blocker use reduced all-cause mortality in all age groups (*P* < 0.001; Figures 4A-1,B-1). Furthermore, RAS inhibitors significantly reduced all-cause mortality in patients aged <80 years and in those aged ≥80 years (*P* < 0.001; Figures 4A-2,A-3). Contrastingly, beta-blocker use significantly reduced all-cause mortality in patients aged <80 years (*P* < 0.001, Figure 4B-2) but not in those aged ≥80 years (*P* = 0.319, Figure 4B-3).

**Figure 5:**
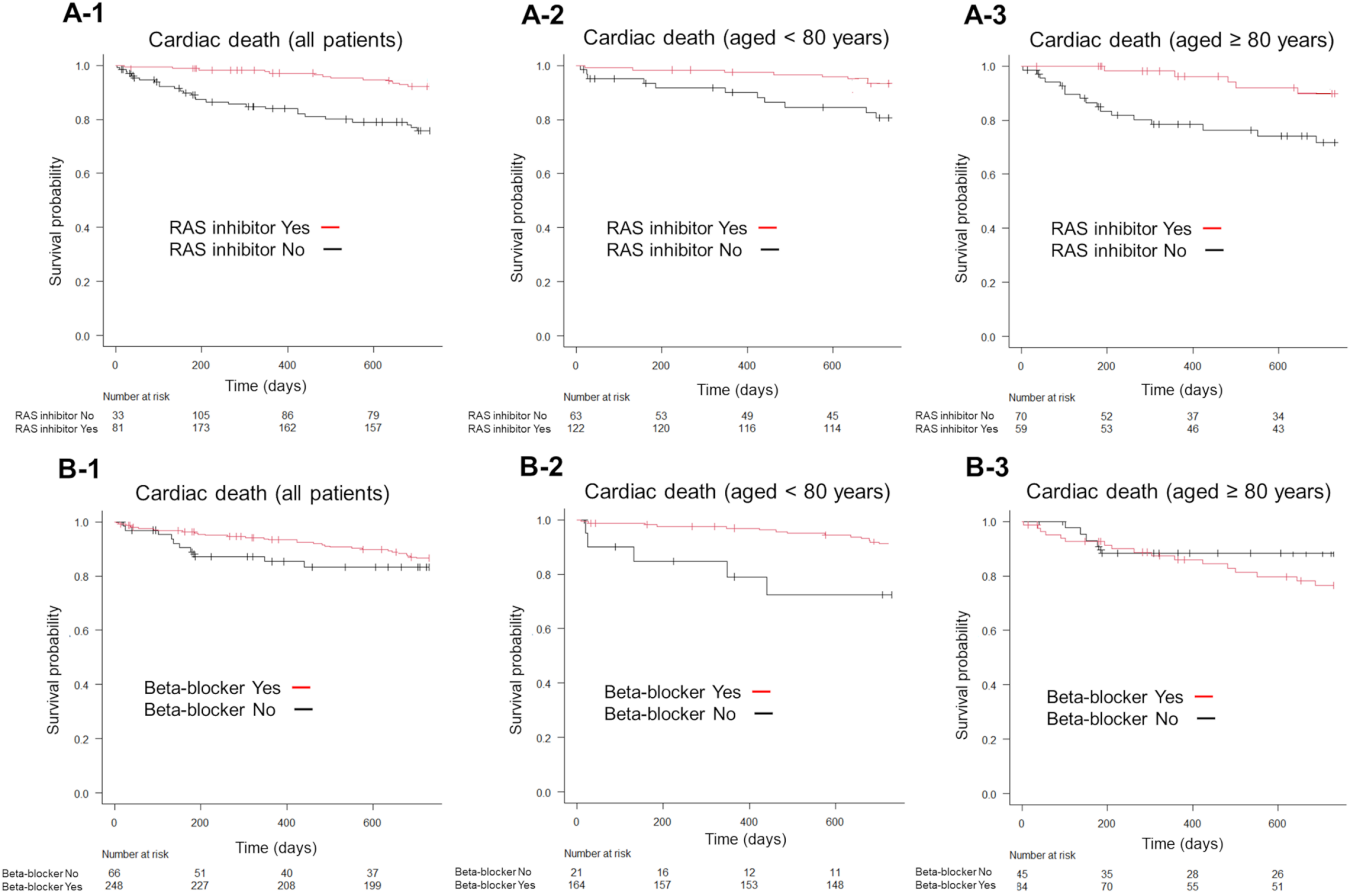
Kaplan–Meier survival curves for the endpoint of cardiac death, according to the GDMT strategy. Patients receiving RAS inhibitors exhibited lower cardiac death than those not receiving RAS inhibitors (**A-1**). Conversely, beta-blocker use did not significantly alter cardiac death rates compared to non-use (**B-1**). The use of RAS inhibitors significantly reduced cardiac death in both patient groups, those aged <80 years and those aged ≥80 years, compared to non-use (**A-2,A-3**). In patients aged <80 years, beta-blocker use was associated with lower cardiac death-related mortality than non-use (**B-2**), whereas in patients aged ≥80 years, beta-blocker use did not show a significant effect compared to non-use (**B-3**). GDMT, guideline directed medical therapy; RAS, renin-angiotensin system.

On the other hand, RAS inhibitor use significantly reduced the incidence of cardiac death (*P* < 0.001; Figure 5A-1), while beta-blocker use showed no significant differences in all age groups (*P* = 0.28; Figure 5B-1). Furthermore, RAS inhibitors significantly reduced cardiac death in patients aged <80 years and in those aged ≥80 years (*P* < 0.001; Figures 5A-2,A-3). Contrastingly, beta-blocker use significantly reduced cardiac death in patients aged <80 years (*P* < 0.001, Figure 5B-2) but not in those aged ≥80 years (*P* = 0.246, Figure 5B-3).

## 4 Discussion

This multicenter prospective cohort study showed the following: (1) RAS inhibitor use was associated with reduced all-cause mortality in patients with HFrEF of all ages; (2) beta-blocker use had an interaction with age; and (3) beta-blocker use was associated with lower all-cause mortality in patients with HFrEF aged <80 years, but not in those aged ≥80 years.

RAS inhibitors appear to reduce all-cause mortality at all ages (24); however, beta-blockers do not appear to reduce all-cause mortality beyond a certain age in older patients with HFrEF. In the Study of the Effects of Nebivolol Intervention on Outcomes and Rehospitalisation in Seniors with Heart Failure, involving a large cohort of patients aged ≥70 years with a history of HF, the beta-blocker nebivolol reduced the composite of all-cause mortality and HF rehospitalization but not all-cause mortality alone (25). In the observational Korean Acute Heart Failure registry, the combined use of beta-blockers and RAS inhibitors reduced all-cause mortality even in patients aged ≥80 years. However, beta-blocker use alone did not reduce all-cause mortality (26). In the Organized Program to Initiate Lifesaving Treatment in Hospitalized Patients with Heart Failure registry, beta-blocker use was not associated with improved survival in patients aged ≥75 years (27). Furthermore, in those aged ≥80 years, the benefit of beta-blockers decreases with age, and its use is not associated with a decreased risk of cardiac death or rehospitalization (28). These findings suggest that beta-blockers decrease in efficacy after a certain age. In a study involving the aging Japanese population, we identified the need to clarify the effectiveness of beta-blockers in older adults and the age threshold at which they cease to be effective. Therefore, we used data from the Kochi YOSACOI study, a registry study of patients hospitalized for acute compensated HF in a region of Japan with a large older adult population (18, 20, 29).

Herein, SHAP was calculated using ML gradient boosting trees, and SHAP interaction values were calculated to quantitatively study the interaction effect between age and beta-blockers (30). This method can identify potentially ambiguous variable interactions and data patterns, and it revealed that beta-blocker efficacy was limited to patients aged <80 years. The age cutoffs in previous reports (70, 75, or 80 years) were determined by the mean age of the patient group studied or by the investigator's rule of thumb (25–28, 31). Therefore, the basis for their decision was ambiguous. In the implemented ML approach, age is considered an explanatory variable, and threshold values and groupings are automatically implemented based on the data to improve generalization performance. By using the ML technique in addition to traditional statistical models, we could analyze the efficacy of beta-blockers in Japanese patients up to the age of 80 years.

Several reasons may account for the reduced usefulness of beta-blockers in older patients with HFrEF. Decreased responsiveness to drugs in older patients is mainly attributed to pharmacodynamic changes associated with aging, such as changes in receptor density and sensitivity, endocrine activation, and autonomic nervous system changes (28). In particular, the sympathetic nervous system is weakened in older patients (24), which may counteract the effects of beta-blockers. In this study, beta-blockers significantly reduced the incidence of cardiac death in patients aged <80 years but did not reduce all-cause mortality in patients aged ≥80 years (Figures 4E-2,F-2). Furthermore, in this study, 5.4% of deaths in patients aged <80 years were due to noncardiovascular causes, whereas the proportion of deaths due to noncardiovascular causes increased to 15.5% in patients aged ≥80 years (Supplementary Material S1). We also compared the characteristics of patients aged <80 years and those aged ≥80 years (Supplementary Material S2) and found significant differences in physical findings, comorbidities, discharge laboratory data, and discharge medications. Our results suggest that management other than HF treatment is also important because noncardiac risk factors have more influence on the death of older patients with HF.

The present study has some limitations. First, despite adjusting for important covariates, owing to the observational nature of the study, confounding factors might have influenced the study results. Furthermore, we could not rule out that unmeasured factors influenced the results. To accurately identify whether beta-blockers are ineffective in older patients with HF, randomized controlled trials are required. Second, we did not assess post-discharge cognitive impairment and medication adherence. Cognitive impairment and medication adherence affect the life expectancy of patients with HF (29), and investigating these issues could be a valuable avenue for future research in this area. Third, this study did not consider the relationship between drug dose and prognosis. Although drug dose is not associated with prognosis in older patients with HF (28), this should be considered in future studies. Finally, although this was a multicenter collaborative study, the all-cause mortality within 2 years was only 80 cases, which limited the number of factors that could be included in the multivariate analysis, and it is possible that confounding could not be adequately controlled. In the future, larger-scale and long-term multicenter collaborative studies should be conducted.

## 5 Conclusion

RAS inhibitors effectively prevented all-cause mortality in patients with HFrEF of all ages, whereas beta-blockers had varying effects according to the patient's age. Therefore, the choice of beta-blockers and RAS inhibitors is more important in older patients with HFrEF.

## Data Availability

The original contributions presented in the study are included in the article/Supplementary Material, further inquiries can be directed to the corresponding author.
