# Supplementary material for: Effects of renin-angiotensin system inhibitor and beta-blocker use on mortality in older patients with heart failure with reduced ejection fraction in Japan
Source: Front Cardiovasc Med. 2024 May 31;11:1377228. doi: 10.3389/fcvm.2024.1377228 (PMC11177874; doi:10.3389/fcvm.2024.1377228)
Supplement: Supplementary file 1 [file Datasheet1.docx]

Supplementary Material

# Supplementary Data. Mortality factors by age

|  | **Age < 80 years** | **Age ≥ 80 years** |
| --- | --- | --- |
| Total patients, n  All-cause mortality, n (%)  Cardiac death, n (%)  Cardiovascular death other than cardiac death, n (%)  Non-cardiovascular deaths, n (%)  Unknown n, (%) | 185  30 (16.2)  19 (10.3)  0  10 (5.4)  1 (0.5) | 129  50 (38.8)  22 (17.1)  4 (3.1)  20 (15.5)  4 (3.1) |
