# Supplementary material for: Effects of renin-angiotensin system inhibitor and beta-blocker use on mortality in older patients with heart failure with reduced ejection fraction in Japan
Source: Front Cardiovasc Med. 2024 May 31;11:1377228. doi: 10.3389/fcvm.2024.1377228 (PMC11177874; doi:10.3389/fcvm.2024.1377228)
Supplement: Supplementary file 2 [file Datasheet2.docx]

Supplementary Material

1. Supplementary Data. Characteristics of patients aged < 80 years and aged ≥ 80 years

| **Variable** | **All patients**  **(n = 314)** | **Aged < 80**  **(n = 185)** | **Age ≥ 80**  **(n = 129)** | **P-value** |
| --- | --- | --- | --- | --- |
| Sex, female | 118 (37.6) | 58 (31.4) | 60 (46.5) | 0.009 |
| BMI (kg/m^2^)  GNRI  NYHA class III/IV at discharge  Prior HF-related admission  **Etiology of heart failure exacerbation**  Ischemic heart disease  Valvular heart disease  Cardiomyopathy  Hypertension  **Comorbidities**  Hypertension  Diabetes mellitus  Dyslipidemia  Atrial fibrillation/flutter  Old myocardial infarction  COPD  Bronchial asthma  Cerebrovascular accident  **Laboratory data at discharge**  BNP, pg/mL  eGFR, mL/min/1.73 m^2^  Sodium, mEq/L  LVEF, %  **Medication at discharge**  RAS inhibitors  Beta-blockers  MRAs  Loop diuretics  Thiazide diuretics  Calcium channel blockers  Digitalis  Tolvaptan  Anticoagulant agents  **Clinical Outcomes**  All-cause mortality within 2 years  Cardiac mortality within 2 years | 20.6 (18.3, 23.2)  92.2 (83.6, 100.0)  16 (5.1)  111 (35.4)  118 (37.6)  30 (9.6)  101 (32.2)  19 (6.1)  197 (62.7)  110 (35.0)  148 (47.1)  130 (41.4)  72 (22.9)  28 (8.9)  16 (5.1)  57 (18.2)  378.8 (196.2, 621.1)  46.3 (32.6, 62.7)  139 (137, 141)  30 (25, 35)  181 (57.6)  248 (79.0)  142 (45.2)  290 (92.4)  18 (5.7)  57 (18.2)  1 (0.3)  90 (28.7)  156 (49.7)  80 (25.5)  41 (13.1) | 21.5 (19.2, 24.4)  95.6 (88.0, 104.0)  9 (4.9)  54 (29.2)  63 (34.1)  8 (4.3)  72 (38.9)  13 (7.0)  108 (58.4)  71 (38.4)  91 (49.2)  66 (35.7)  34 (18.4)  12 (6.5)  7 (3.8)  30 (16.2)  323.0 (171.3, 540.8)  49.9 (35.3, 66.5)  139 (137, 141)  29 (23, 34)  122 (65.9)  164 (88.6)  84 (45.4)  171 (92.4)  10 (5.4)  31 (16.8)  0 (0.0)  50 (27.0)  95 (53.0)  30 (16.2)  19 (10.3) | 19.3 (17.4, 21.6)  86.9 (78.8, 94.5)  7 (5.4)  57 (44.2)  55 (42.6)  22 (17.1)  29 (22.5)  6 (4.7)  89 (69.0)  39 (30.2)  57 (44.2)  64 (49.6)  38 (29.5)  16 (12.4)  9 (7.0)  27 (20.9)  451.5 (242.3, 773.5)  41.2 (31.5, 57.8)  138 (137, 141)  32.5 (27, 37)  59 (45.7)  84 (65.1)  58 (45.0)  119 (92.2)  8 (6.2)  26 (20.2)  1 (0.78)  40 (31.0)  61 (47.3)  50 (38.8)  22 (17.1) | <0.001  <0.001  0.99  0.008  0.126  <0.001  0.002  0.475  0.059  0.15  0.422  0.015  0.029  0.106  0.297  0.301  0.006  0.01  0.4  <0.001  <0.001  <0.001  0.99  0.99  0.81  0.46  -  0.45  0.493  <0.001  0.09 |

Abbreviations: BMI, body mass index; BNP, B-type natriuretic peptide; COPD, chronic obstructive pulmonary disease; eGFR, estimated glomerular filtration rate; GNRI, Geriatric Nutritional Risk Index; HF, heart failure; LVEF, left ventricular ejection fraction; MRA, mineralocorticoid receptor antagonist; NYHA, New York Heart Association; RAS, renin-angiotensin system

Data are shown as median [interquartile range] or n (%)
